# Lived experiences of post-traumatic growth in patients with acute type A aortic dissection: A qualitative study

**DOI:** 10.1371/journal.pone.0358013

**Published:** 2026-10-15

**Authors:** Ping Zhang, Chun Liu, Anni Hu, Di Wang, Xuerui Wang, Qing Zhou, Yunyan Su

**Affiliations:** 1 Department of Cardiac Surgery, Nanjing Drum Tower Hospital, Affiliated Hospital of Medical School, Nanjing University, Nanjing, Jiangsu, China; 2 Department of Emergency, The First Affiliated Hospital of Anhui Medical University, Hefei, Anhui, China; 3 Department of General Surgery, Nanjing Drum Tower Hospital, Affiliated Hospital of Medical School, Nanjing University, Nanjing, Jiangsu, China; PLOS: Public Library of Science, UNITED KINGDOM OF GREAT BRITAIN AND NORTHERN IRELAND

## Abstract

**Introduction:**

Acute type A aortic dissection is a life-threatening cardiovascular emergency characterized by sudden onset, severe pain, and high mortality, exerting profound physical and psychological burdens on affected individuals. While negative psychological sequelae predominate in most cases, a subset of patients demonstrates remarkable resilience, manifesting post-traumatic growth—a positive psychological transformation emerging in the aftermath of trauma. This qualitative study aimed to investigate the lived experiences of post-traumatic growth among Acute type A aortic dissection survivors, shedding light on this understudied dimension of their recovery trajectory.

**Methods:**

A qualitative study was conducted using a phenomenological approach. Twenty patients diagnosed with Acute type A aortic dissection were recruited through purposive sampling from a tertiary hospital in China. Data were collected via in-depth, face-to-face semi-structured interviews and analyzed using Colaizzi’s seven-step method to extract key themes and sub-themes.

**Results:**

Three overarching themes and eight sub-themes were identified, reflecting the multidimensional nature of post-traumatic growth in Acute type A aortic dissection patients: Reevaluating Life Priorities (reordering life priorities, valuing life), Behavioral Transformation (improving unhealthy habits, enhancing treatment adherence), and Enhanced Social Connections (strengthened family ties, gratitude for social support, reintegration into family and society, altruistic behavior).

**Conclusions:**

This study elucidates three core dimensions of post-traumatic growth in survivors of acute type A aortic dissection: life priority reassessment, adoption of positive health behaviors, and enhanced social connectedness. The findings underscore the critical role of guided self-reflection, supported behavior change, and robust family-social support systems in facilitating psychological recovery. We recommend integrating interventions focused on post-traumatic growth, such as psychological counseling and structured family support, into standard cardiac rehabilitation protocols to promote holistic recovery. Together, these insights provide a theoretical and practical foundation for developing tailored psychosocial interventions for this patient population.

## Introduction

Experiencing a cardiac event or living with cardiovascular disease often imposes significant psychological burdens, with many patients perceiving such events as traumatic [1]. Research indicates that approximately 75% of individuals who have experienced a cardiac incident regard it as psychologically traumatic [2]. A recent meta-analysis of patients who experienced cardiac arrest reported pooled prevalence rates of 19% for depression, 26% for anxiety, and 20% for post-traumatic stress disorder (PTSD) during the early post-event period [3]. These persistent psychological symptoms—including intrusive thoughts, hyperarousal, sleep disturbances, and emotional numbing—can substantially impair quality of life, reduce treatment adherence, and hinder physical recovery [4]. Notably, PTSD not only arises as a consequence of cardiac illness but also constitutes a potential risk factor for future cardiovascular events, underscoring a complex and bidirectional relationship between psychological trauma and cardiovascular health [5].

While many patients respond to major illness with negative psychological outcomes, a significant proportion exhibit resilience and report positive psychological changes [6,7]. This phenomenon, known as post-traumatic growth (PTG), refers to positive psychological transformation following adversity, particularly in areas such as self-perception, interpersonal relationships, and life philosophy [8]. For instance, a study involving 82 survivors of myocardial infarction and coronary artery bypass graft surgery found that 71.2% reported experiencing PTG [9]. Rather than being a linear process, PTG unfolds dynamically through cognitive restructuring, emotional regulation, and meaning-making. It reflects an active psychological adaptation that enables individuals to derive meaning from trauma, strengthen psychological resilience, and enhance their overall well-being [10]. In recent years, PTG has garnered increasing scholarly attention as a positive psychological response to adversity [11]. PTG has been documented in individuals coping with a wide range of stressors, including cancer, terrorist attacks, and the COVID-19 pandemic, underscoring its broad applicability across diverse traumatic contexts [12–14].

Acute type A aortic dissection (ATAAD) is among the most catastrophic and rapidly progressive cardiovascular emergencies, associated with extremely high mortality. Epidemiological data from Stockholm County report an annual incidence of acute aortic dissection of approximately 4.1 per 100,000, of which nearly two-thirds are ATAAD, with a 30-day mortality of approximately 28% [15]. The clinical onset of ATAAD is frequently marked by sudden and excruciating chest or back pain, symptoms that patients commonly describe as a “near-death sensation” or the “worst pain imaginable” [4]. The abrupt onset, overwhelming physical distress, and life-threatening nature of the condition may result in profound psychological trauma. This makes ATAAD survivors a particularly relevant population for exploring not only post-traumatic stress but also the possibility of psychological growth.

Of note, research on PTG has advanced considerably across various critical illness populations. For example, qualitative studies involving cancer survivors have revealed that PTG manifestations can encompass unique aspects such as health behavior changes, which are often categorized within the dimension of “new possibilities” [16]. Similarly, a meta-synthesis of qualitative evidence in young and middle-aged stroke patients has proposed a three-phase developmental framework—encompassing trauma and stress, adjustment and reconstruction, and eventual growth—thereby elucidating the progression of PTG across physical, psychological, and social domains [17]. These findings collectively suggest that clinical features, recovery trajectories, and patient demographics may contribute to disease-specific PTG experiences.

In contrast, the experience of ATAAD is marked by several distinct characteristics: a sudden and severe onset, frequent requirement for emergency surgery, and a complex, often uncertain recovery pathway. These features are likely to shape patients’ cognitive appraisal of the trauma, their meaning-making processes, and the particular manifestations of PTG. As a result, the PTG experience in ATAAD survivors may not be fully captured by theoretical models derived from other conditions, such as cancer or stroke.

Given these distinctions, a qualitative approach is particularly well-suited to explore the unique contours of PTG in this population. Although PTG has gained increasing attention in cardiovascular research, most studies to date have employed quantitative methodologies [18], often utilizing standardized instruments such as the Posttraumatic Growth Inventory to assess the presence and correlates of PTG [19]. While such approaches yield valuable generalizable insights, PTG remains an inherently subjective and deeply personal phenomenon that may not be fully captured through structured assessments alone. Thus, qualitative inquiry is essential to uncover the nuanced lived experiences and contextual factors underlying PTG in ATAAD survivors.

This study aims to explore the lived experiences of PTG among patients recovering from ATAAD surgery. By examining how individuals perceive, interpret, and articulate their growth following this life-threatening event, our research seeks to provide a deeper understanding of PTG in the context of acute cardiovascular trauma. Ultimately, these insights may inform more holistic and psychologically sensitive clinical care for this vulnerable population.

## Materials and methods

### Participants and setting

From April 2024 to December 2024, a purposive sampling strategy was employed to recruit patients diagnosed with ATAAD who had undergone surgical treatment at the Department of Cardiac Surgery in eastern China. This sampling method is particularly well-suited for qualitative research, as it enables the selection of participants who possess rich, relevant, and diverse experiences related to the phenomenon under investigation [20].

To enhance the diversity and representativeness of the sample, key demographic factors such as gender, age, educational background, marital status, and occupation were carefully considered during recruitment. The final sample size was guided by the principle of data saturation [21]. Saturation was assessed using the constant comparative method, with data collection and analysis proceeding concurrently. The operational criterion for saturation was set as three consecutive interviews (16–18) yielding no new codes relevant to the core themes. This was subsequently confirmed by two additional interviews (total n = 20), establishing that the sample was sufficient to capture the core dimensions of the phenomenon.

Inclusion criteria were as follows: first-time diagnosis of ATAAD with surgical intervention performed during the current hospitalization; clear consciousness, the ability to communicate effectively, and no history of severe cognitive impairment or major psychiatric disorders; provision of informed consent and voluntary participation.

Exclusion criteria included: the occurrence of major traumatic events within the six months preceding the study; refusal to participate or withdrawal from the study at any point.

In total, 20 patients meeting the criteria participated in the study. Their basic demographic characteristics are presented in Table 1.

**Table 1:** Demographic data of participants in the study.

Participant number	Gender	Age Ranges	Educational Status	Marital status	Occupation
P1	Male	60-69	Senior high school	Married	Worker
P2	Female	30-39	University	Married	Employee
P3	Male	60-69	Senior high school	Married	Freelance career
P4	Male	40-49	Senior high school	Married	Employee
P5	Male	50-59	Senior high school	Married	Employee
P6	Female	70-79	Senior high school	Married	Retired
P7	Female	60-69	Primary school	Married	Retired
P8	Male	40-49	Junior high school	Married	Freelance career
P9	Male	40-49	Junior high school	Married	Freelance career
P10	Male	60-69	Primary school	Married	Worker
P11	Male	30-39	Junior high school	Married	Worker
P12	Male	40-49	Junior high school	Married	Employee
P13	Female	70-79	Primary school	Single	Retired
P14	Male	30-39	Junior high school	Single	Freelance career
P15	Male	50-59	Junior high school	Married	Retired
P16	Male	40-49	Senior high school	Married	Employee
P17	Female	50-59	Junior high school	Married	Retired
P18	Male	50-59	Junior high school	Married	Employee
P19	Male	50-59	Primary school	Married	Retired
P20	Female	60-69	Primary school	Married	Retired

### Data collection

A phenomenological approach was employed to guide data collection through semi-structured, in-depth, face-to-face interviews. All interviews were conducted in a designated follow-up room within the Department of Cardiac Surgery. Initially, the research team collaborated with clinical nursing staff to identify eligible participants among postoperative cardiac surgery patients. Subsequently, potential participants were further screened and invited based on the established inclusion and exclusion criteria. This multi-step recruitment process, along with detailed documentation of clinical pathways and a systematic characterization of the care setting, strengthens the contextual depth of the data. This, in turn, facilitates the transferability of the findings to other settings sharing similar clinical and cultural contexts.

Prior to each interview, researchers built rapport with participants, clearly explained the purpose of the study, assured them of confidentiality and privacy, and obtained written informed consent. Interviews were conducted in a quiet, private setting conducive to open and relaxed communication, which facilitated the collection of authentic and meaningful data.

Two primary instruments were used for data collection. The first was a demographic questionnaire capturing key participant characteristics, including age, gender, educational level, marital status, and occupation. The second was a semi-structured interview guide, developed based on the study objectives, a review of relevant literature, and the theoretical framework of PTG as proposed by Tedeschi and Calhoun [8]. This framework, which conceptualizes PTG across five domains (personal strength, new possibilities, relating to others, appreciation of life, and spiritual change), was selected for its specific utility in capturing the processes of positive psychological adaptation following life-threatening events such as ATAAD.

To ensure the clarity, relevance, and sensitivity of the interview questions, a pilot study was conducted with five ATAAD patients who met the inclusion criteria but were not included in the final sample. These preliminary interviews allowed the researchers to assess the comprehensibility, emotional appropriateness, and logical sequence of the questions. Based on feedback, minor revisions were made—for example, simplifying complex language, reordering items for improved flow, and incorporating more open-ended prompts to encourage deeper personal reflection.

The finalized interview guide focused on several core themes related to PTG, with specific questions detailed in Table 2. Each interview lasted approximately 30–50 minutes, was audio-recorded with participants’ permission, and supplemented by field notes capturing non-verbal cues and contextual observations. Researchers engaged in active listening and used probing techniques when appropriate to elicit deeper insights. All interviews were transcribed verbatim for subsequent thematic analysis.

**Table 2:** Interview guideline: Open questions.

No.	Questions
1	Could you share your experience of falling ill and undergoing surgery?
2	During your illness and treatment, what emotional and psychological changes did you experience?
3	What challenges and pressures did you face during this process? How did you cope with them?
4	After your illness, have there been any changes in the emotional attitudes of your family, relatives, or friends toward you?
5	What positive changes have you noticed as a result of this experience?
6	What concerns do you currently have, and what are your expectations or plans for the future?
7	If you were to meet a patient with a similar experience, how would you encourage or advise them?
8	Is there anything else you would like to add or share?

### Data analysis

All interview data were managed and organized using NVivo 12 software (QSR International (Americas) Inc, Burlington, MA, USA). To ensure accuracy and completeness, the interview transcripts were independently reviewed and verified by two researchers. Data analysis followed Colaizzi’s seven-step phenomenological method, which provides a rigorous and systematic approach to exploring participants’ lived experiences. This process involved repeated reading of the transcripts, identification of significant statements, coding of relevant data, and the development of thematic structures.

Data were analyzed using Colaizzi’s phenomenological approach, proceeding through three primary phases: initial coding, theme development, and validation.

During the initial coding phase, interviews were transcribed verbatim and repeatedly reviewed to ensure immersion in the data. Significant statements were systematically identified, and their underlying meanings were formulated, resulting in 135 distinct statements and 8 condensed meaning units.

In the theme development phase, these meaning units were grouped into preliminary thematic categories based on conceptual similarities. Through iterative research team discussions, the categories were refined and synthesized into a coherent thematic structure, culminating in three overarching themes and eight sub-themes that captured essential aspects of participants’ PTG experiences.

Finally, during the validation phase, the thematic structure was presented to participants for member checking. The majority affirmed that the findings accurately reflected their lived experiences, thereby strengthening the trustworthiness of the analytic process.

### Trustworthiness

The trustworthiness of this study was evaluated using Guba and Lincoln’s framework, with emphasis placed on the strategies most essential for ensuring methodological rigor and interpretive validity [22].

Credibility was strengthened through two principal approaches. Member checking served as a primary validation mechanism, in which participants confirmed that selected transcripts and emergent themes accurately reflected their experiences. In addition, prolonged engagement with the data—through repeated reading of transcripts and reflective dialogue with participants—ensured that interpretations remained grounded in lived realities. Interviews were conducted using a semi-structured guide developed from a comprehensive literature review, balancing consistency in exploring PTG dimensions with flexibility for individual narrative expression.

Transferability was supported mainly by providing rich, contextual descriptions along with illustrative participant quotations. These elements furnish sufficient detail to allow readers to assess the potential applicability of the findings to comparable clinical settings or populations.

Dependability was achieved through systematic documentation of the research process, including the development of interview protocols, data collection procedures, and analytical decisions. Regular research team meetings further enhanced interpretive consistency. Confirmability was strengthened by reflexive practices and maintenance of an audit trail, ensuring that findings emerged directly from the data rather than researcher presuppositions.

Together, these approaches demonstrate a commitment to research rigor, with member checking and thick description serving as cornerstone strategies for ensuring credibility and transferability, respectively.

### Ethics

This study was conducted in accordance with the principles of the Declaration of Helsinki and was approved by the Ethics Committee of a tertiary hospital in eastern China (Approval Code: 2024-089-02; Approval Date: March 11, 2024). All participants provided written informed consent after receiving a detailed information sheet explaining the study’s purpose, procedures, potential risks, and their rights.

To protect participants’ rights, they were explicitly advised of their unconditional right to withdraw at any time without penalty, including options to pause, skip questions, or end the interview. Confidentiality was ensured through the removal of all identifiable personal information from transcripts, the use of pseudonyms, and the omission or alteration of potentially identifying details in publications. All research data were stored on a secure, password-protected server accessible only to the principal investigators.

Trained researchers conducted interviews using a sensitive, empathetic, and non-judgmental approach to minimize distress. Participants were encouraged to take breaks as needed, and a support protocol was established to promptly pause sessions and provide access to a clinical psychologist if significant emotional distress arose. No such interventions were required during the study.

## Results

Thematic analysis revealed three interrelated overarching themes and eight corresponding sub-themes, capturing the dynamic process of PTG in patients following ATAAD surgery. These themes—Reevaluating Life Priorities, Behavioral Transformation, and Enhanced Social Connections—collectively reflect a progression from cognitive restructuring through behavioral adaptation to deepened interpersonal engagement. As summarized in Table 3, the thematic structure includes: Reevaluating Life Priorities, comprising Reordering Life Priorities and Valuing Life; Behavioral Transformation, consisting of Improving Unhealthy Habits and Enhancing Treatment Adherence; and Enhanced Social Connections, which includes Strengthened Family Ties, Gratitude for Social Support, Reintegration into Family and Society, and Altruistic Behavior.

**Table 3:** Themes, sub-themes, and representative quotes from interview data.

Theme	Sub-theme	Examples of initial coding
Reevaluating Life Priorities	Reordering Life Priorities	Realizing health comes first Putting family before career Learning to take care of oneself
	Valuing Life	Cherishing each day more Feeling lucky to be alive Understanding that life can change in a second
Behavioral Transformation	Improving Unhealthy Habits	Giving up old bad habits Paying more attention to diet Making rest a daily priority
	Enhancing Treatment Adherence	Being strict with medication Never skipping check-ups Taking blood pressure seriously
Enhanced Social Connections	Strengthened Family Ties	Becoming closer with loved ones Feeling warmth from family Appreciating family care deeply
	Gratitude for Social Support	Touched by medical staff’s kindness Thankful for friends’ support Remembering others’ help
	Reintegration into Family and Society	Wanting to spend more time with family Planning a slower return to work Living a more relaxed life
	Altruistic Behavior	Sharing recovery experience Encouraging other patients Wanting to give back with love and kindness

### Theme 1: Reevaluating life priorities

This theme elucidates how participants reexamined core values and developed renewed life appreciation following near-death ATAAD experience, reflecting cognitive restructuring within the PTG framework.

#### Reordering life priorities.

Analysis revealed a fundamental shift in life priorities post-ATAAD, characterized by transition from professional ambitions to personal health and family relationships. This reprioritization constitutes a key manifestation of cognitive restructuring in PTG, wherein traumatic events precipitate core value reassessment.

Participants reported increased emphasis on health and family over occupational pursuits. One stated:

“Before this illness, I focused on work and making money. Now I realize that being healthy and present for my family matters more.” (P5)

Another corroborated: “I always put work first. This made me see that without health, nothing else matters.” (P2)

Some described tension between new health awareness and existing responsibilities. A participant explained:

“I know I need to slow down, but I have financial obligations. It’s a constant balance between what’s good for my health and what life requires. I’m trying to find a middle ground.” (P11)

This illustrates the negotiation between ideal recovery and practical constraints.

Reprioritization magnitude varied considerably among individuals, underscoring the individualized nature of PTG and diverse integration of life-threatening experiences into personal value systems.

#### Valuing life.

ATAAD survival precipitated existential awakening, marked by enhanced life appreciation and redefined purpose. This represents a core PTG dimension where mortality confrontation facilitates deeper existential engagement.

Many evolved from taking life for granted to perceiving daily existence as meaningful. One described:

“Waking up after surgery, I realized how close I came to death—that changed everything.” (P8)

This mortality awareness catalyzed more grateful worldviews.

Some framed appreciation philosophically:

“The illness came so suddenly. It felt like fate’s reminder that life is precious.” (P5)

Suggesting meaning attribution may facilitate psychological integration.

Value transformation varied emotionally. While many expressed serene gratitude, others described motivated outlooks:

“I used to take life for granted. Now every day feels like a gift.” (P14)

This heterogeneity illustrates life valuation as a multidimensional construct reflecting diverse PTG pathways.

### Theme 2: Behavioral transformation

This theme reflects the psychological and behavioral adjustments participants made in response to their health crisis, demonstrating a proactive approach to recovery and self-care.

#### Improving unhealthy habits.

Participants consistently reported making decisive changes to long-standing unhealthy behaviors following their ATAAD diagnosis, reflecting a regained sense of control and self-efficacy. This behavioral activation is a hallmark of proactive coping within the PTG framework.

A strong motivation to eliminate health risks was frequently described. As one participant stated:

“I’d smoked and drank for 30 years. This illness made me quit for good.” (P3)

This illustrates how a life-threatening diagnosis can provide a compelling impetus for sustained behavioral change.

Changes extended to proactive, health-promoting routines. One participant explained:

“I used to skip meals and eat junk food. Now I watch my diet and rest more.” (P4)

This shift underscores a more mindful and agentic relationship with health.

Some participants noted challenges in maintaining new habits:

“When everyone around me is indulging in eating and drinking without restraint, I find it hard to stick to a healthy lifestyle. Sometimes I can’t help but give in, and I’m filled with guilt afterward” (P2)

Recognition of these challenges presents a realistic picture of behavioral growth as an ongoing process.

#### Enhancing treatment adherence.

Participants demonstrated significantly improved adherence to medical regimens post-ATAAD, driven by acute vulnerability awareness and resolve to prevent recurrence.

A heightened sense of personal responsibility was commonly observed. One participant articulated:

“I promised myself to take my meds on time and go for regular check-ups.”(P10)

Improved adherence was associated with better understanding of bodily signals:

“I used to ignore symptoms, but now I follow all the doctor’s instructions.”(P17)

Vigilance was often rooted in identifying a specific cause:

“I know my high blood pressure played a role. This time, I’ll control it carefully.” (P7)

Some acknowledged the psychological burden

“It’s exhausting to constantly be the ‘responsible patient,’ always thinking about numbers, pills, and restrictions. You can never truly forget what happened.” (P13)

This reveals the dual nature of behavioral transformation as both empowering and demanding.

### Theme 3: Enhanced social connections

This theme reflects how participants developed deeper social bonds and strengthened connections with others, characterized by gratitude, emotional closeness, and a desire to contribute to the well-being of others.

#### Strengthened family ties.

The experience of ATAAD often served as a catalyst for deepening family relationships. Many participants emerged with renewed appreciation for family bonds, characterized by emotional closeness and gratitude for support received, illustrating the interpersonal dimension of PTG.

The consistent presence of family during illness was foundational. One participant reflected:

“My wife stayed by my side every day. We’ve grown so much closer.” (P1)

This shows how caregiving during critical illness fosters deeper intimacy.

The illness also prompted reconnections with wider family networks. As one participant shared:

“My children came from far away to see me. That meant a lot.” (P9)

Here, the crisis strengthened intergenerational bonds and reinforced belonging.

Receiving care sometimes led to positive role reversals. One participant expressed:

“My brother took care of me, even though it wasn’t easy. I’m really grateful.” (P12)

This suggests PTG can involve constructive role renegotiation.

Some participants noted that increased closeness brought complexity:

“You feel immense pressure to ‘not waste’ this second chance, to be a better family person. It’s beautiful but also heavy—the expectations can feel burdensome.” (P15)

Acknowledging this adds depth to understanding family bond intensification.

#### Gratitude for social support.

Participants expressed profound gratitude for support from healthcare providers and personal networks, reinforcing their sense of being valued.

Professional care beyond formal duties was impactful. One participant recalled:

“A nurse stayed after her shift just to check on me. That kindness gave me strength.” (P20)

This highlights how empathetic care affirms patient worth.

Support from friends reaffirmed social identity:

“My friends visited, encouraged me, and even helped my family. I’m lucky to have them.” (P15)

This demonstrates how support buffers against identity erosion.

Gratitude toward medical teams motivated recovery:

“The director performed surgery on me overnight. He was exhausted. I owe him my life.” (P19)

This shows how gratitude can become a moral obligation driving rehabilitation.

Some felt pressure to be worthy of support:

“The immense kindness can feel overwhelming. You feel pressure not to let people down, to make their effort ‘worth it’ by being the perfect recovery story.” (P18)

This nuance reveals that gratitude can coexist with burden.

#### Reintegration into family and society.

Following initial recovery, participants demonstrated a deliberate intent to reintegrate into social and familial roles, guided by a transformed perspective that prioritized purposeful engagement over pre-illness obligations.

Many sought to deepen family relationships through presence rather than instrumental contributions. As one participant described:

“I want to spend more time with my grandchildren and enjoy life.” (P6)

This shift highlights a qualitative transformation from a provider role to relational engagement.

In the occupational domain, participants often planned to return to work with self-imposed boundaries to safeguard their health and personal life. One stated:

“I’ll go back to work, but I won’t push myself like before.” (P16)

This reflects the development of empowered boundary-setting as part of PTG.

Reintegration was not without challenges. Some acknowledged the difficulty of implementing new perspectives in unchanged environments. A participant explained:

“Changing is one thing, but returning to the same old expectations is another. There’s a clash between who I’ve become and the ‘slot’ the world has for me.” (P11)

This underscores reintegration as an ongoing negotiation between internal transformation and external structures.

#### Altruistic behavior.

Many participants expressed a strong desire to “give back” by supporting others facing similar health challenges, representing an advanced stage in the PTG trajectory.

For some, this took the form of direct peer support. One participant shared:

“I’ve been sharing my story with other patients. I want to keep helping them.” (P11)

This illustrates the transformation of personal suffering into a prosocial resource, reinforcing the helper’s sense of purpose.

Altruism was often motivated by gratitude and a sense of debt to those who provided support. A participant explained:

“This illness taught me the value of kindness. I want to give back, just like others helped me.” (P18)

Here, altruism serves as a moral response that integrates traumatic experience into a narrative of human connection.

Participants expressed this impulse in varied ways, balancing the desire to help with self-protection. One noted:

“I want to help but must guard my energy. Sometimes talking too much brings back anxiety, so I might give practical advice anonymously.” (P14)

This demonstrates that post-traumatic altruism involves careful negotiation between supporting others and preserving one’s own well-being.

## Discussion

This study explored PTG in patients recovering from ATAAD, a life-threatening cardiovascular emergency characterized by rapid onset, high mortality, and significant psychological distress. Drawing upon the theoretical model proposed by Tedeschi and Calhoun—which conceptualizes PTG across five domains: appreciation of life, personal strength, relating to others, new possibilities, and spiritual change [8]—our findings identified three overarching themes: reevaluation of life priorities, behavioral transformation, and enhanced interpersonal connections. These themes reflect a multidimensional adaptation process that extends beyond physical recovery and contributes to a more holistic understanding of survivorship in ATAAD patients.

One of the most salient findings was a renewed appreciation for life, consistent with the PTG domain “appreciation of life.”The sudden and critical nature of ATAAD forced participants to confront their own mortality, often prompting deep existential reflection. Many described their illness as a “second chance,” which led to increased gratitude, greater attentiveness to everyday experiences, and a heightened desire to live more meaningfully. This pattern of increased gratitude and desire for meaningful living is consistent with findings from cancer survivor research, implying a shared pathway for PTG across severe health crises [23]. While direct references to spirituality were rare, many participants demonstrated a culturally embedded awareness of life’s fragility, implying that spiritual change may manifest in implicit, non-religious forms in East Asian contexts. Clinicians may support this reflective process by incorporating meaning-centered interventions into post-discharge care—such as guided life review, narrative therapy, or gratitude journaling—to promote psychological integration and enhance life appreciation.

In terms of behavioral transformation, participants reported significant lifestyle changes, including smoking cessation, dietary improvements, and greater adherence to medical regimens. These behaviors reflected increased self-efficacy and personal responsibility, aligning with the PTG domains of “personal strength” and “new possibilities.”Importantly, these changes were not externally imposed but rather self-initiated and sustained, suggesting a deeper psychological reorganization. Similar findings have been reported in studies of coronary artery disease and cancer survivors [24,25], where acute health events served as powerful catalysts for behavior change. These results are consistent with the “teachable moment” concept in health behavior theory, underscoring how a life-threatening diagnosis can provide a pivotal opportunity for patients to adopt and maintain healthier behaviors [26]. From a nursing perspective, this phase offers a strategic window for implementing structured health coaching, assisting patients in setting individualized recovery goals, and reinforcing personal strengths through positive feedback—thus promoting autonomy and fostering sustainable health-promoting behaviors.

Social and relational growth emerged as a key dimension of PTG in this study, with participants describing a range of interpersonal changes following their ATAAD experience. Many reported strengthened familial bonds, with greater appreciation for emotional support from family members during hospitalization and recovery. Similar findings have been observed in cancer survivors, where critical illness often leads to re-prioritization of close relationships and heightened emotional expression [27]. Participants also expressed gratitude toward healthcare professionals and broader social networks, consistent with previous literature indicating that perceived social support is a key facilitator of PTG [19,28]. In contrast to studies where informal peer support developed organically (e.g., among breast cancer patients), ATAAD survivors voiced a clear desire for more structured opportunities to connect with others who had undergone similar experiences. This gap may reflect the rarity and sudden onset of ATAAD, which limit opportunities for spontaneous peer bonding during hospitalization. Finally, some participants reported a desire to help others by sharing their recovery stories—a finding that echoes altruistic tendencies reported in trauma survivors across contexts, and that may serve as a form of meaning-making and identity reconstruction [29]. Collectively, these findings align with the PTG domain of “relating to others” and underscore the complex interpersonal transformations that accompany recovery. From a nursing perspective, clinical efforts should focus on engaging families, fostering emotional openness, and developing peer-support mechanisms tailored to the unique trajectory of ATAAD survivors.

Interestingly, although “new possibilities” is a central domain in the PTG framework, it was less prominently represented in our data. Rather than pursuing entirely new life directions, most participants focused on restoring stability within their current familial and social roles [7]. The difference may reflect both the intense physical and psychological demands of ATAAD recovery, and the influence of cultural values—particularly in collectivist societies where maintaining family responsibilities is prioritized over individual reinvention. These results underscore the importance of contextualizing PTG manifestations and avoiding a one-size-fits-all interpretation. Growth may take culturally congruent forms that emphasize continuity and integration, rather than radical lifestyle change.

Finally, although spiritual change was not explicitly discussed by most participants, subtle expressions of existential awareness and mortality salience were evident. This suggests that spiritual transformation may be present, albeit in more implicit or culturally nuanced ways. Future studies may benefit from integrating culturally sensitive tools to better capture this dimension of growth.

In summary, the findings suggest that PTG among ATAAD survivors is shaped through dynamic interactions among traumatic illness experiences, cultural context, and interpersonal relationships. Rather than indicating the absence of distress, growth frequently coexisted with ongoing psychological vulnerability and patients’ efforts to restore stability in everyday life. These findings highlight the importance of integrating psychological and social support into post-ATAAD care. Nursing interventions that promote reflective coping, strengthen personal agency, and foster supportive relationships may facilitate patients’psychological integration of their illness experiences throughout recovery.

## Limitations

Despite its valuable insights, this study has several limitations that should be acknowledged. First, the sample was recruited from a single tertiary hospital in China, which may limit the generalizability of the findings to broader ATAAD populations, particularly those from different cultural or healthcare contexts. Second, the qualitative design, while appropriate for exploring in-depth experiences, does not allow for quantification of PTG levels or identification of statistically significant predictors. Third, although all participants were interviewed prior to discharge to ensure consistency in timing, this cross-sectional snapshot may not capture the evolving nature of PTG, which can continue to develop over time. Finally, the study did not incorporate perspectives from family members or caregivers, whose insights might have provided a more comprehensive understanding of interpersonal growth and support dynamics during recovery. Future studies should consider longitudinal designs, multi-center recruitment, and mixed-method approaches to validate and expand upon these findings.

## Conclusion

This study is among the first to qualitatively explore post-traumatic growth in patients recovering from ATAAD, demonstrating that survival is often accompanied by meaningful psychological and social transformation. By identifying key domains of growth—including redefined life priorities, proactive health behaviors, and strengthened interpersonal relationships—this research extends the application of the PTG framework to a previously underexplored cardiovascular population. These findings underscore the importance of holistic, patient-centered care that supports both physical and psychological recovery, and provide a foundation for developing culturally sensitive psychosocial interventions. Future research should further examine the evolution of PTG over time and explore its broader impact within the recovery ecosystem.
